# Incidence and outcome of prosthetic valve endocarditis after transcatheter aortic valve replacement in the Netherlands

**DOI:** 10.1007/s12471-020-01420-2

**Published:** 2020-04-24

**Authors:** J. Brouwer, F. S. van den Brink, V. J. Nijenhuis, T. N. Vossenberg, R. Delewi, M. S. van Mourik, P. den Heijer, W. Tanis, P. C. Kievit, W. Holvoet, R. S. Hermanides, J. M. ten Berg

**Affiliations:** 1 Department of Cardiology, St Antonius Ziekenhuis, Nieuwegein, The Netherlands; 2 Department of Cardiology, Medisch Centrum Leeuwarden, Leeuwarden, The Netherlands; 3 Department of Cardiology, Amsterdam Universitair Medisch Centrum, locatie AMC, Amsterdam, The Netherlands; 4 Department of Cardiology, Amphia Ziekenhuis, Breda, The Netherlands; 5 Department of Cardiology, Haga Ziekenhuis, The Hague, The Netherlands; 6 Department of Cardiology, Radboud Medisch Centrum, Nijmegen, The Netherlands; 7 Department of Cardiology, Maastricht Universitair Medisch Centrum, Maastricht, The Netherlands; 8 Department of Cardiology, Isala Klinieken, Zwolle, The Netherlands

**Keywords:** Structural heart valve disease, Structural heart intervention, Transcatheter aortic valve replacement, Aortic valve stenosis, Prosthetic valve endocarditis

## Abstract

**Background:**

Transcatheter aortic valve replacement (TAVR) is increasingly being used as an alternative to conventional surgical valve replacement. Prosthetic valve endocarditis (PVE) is a rare but feared complication after TAVR, with reported first-year incidences varying from 0.57 to 3.1%. This study was performed to gain insight into the incidence and outcome of PVE after TAVR in the Netherlands.

**Methods:**

A multicentre retrospective registry study was performed. All patients who underwent TAVR in the period 2010–2017 were screened for the diagnosis of infective endocarditis in the insurance database and checked for the presence of PVE before analysis of general characteristics, PVE parameters and outcome.

**Results:**

A total of 3968 patients who underwent TAVR were screened for PVE. During a median follow-up of 33.5 months (interquartile range (IQR) 22.8–45.8), 16 patients suffered from PVE (0.4%), with a median time to onset of 177 days (IQR 67.8–721.3). First-year incidence was 0.24%, and the overall incidence rate was 0.14 events per 1000 person-years. Overall mortality during follow-up in our study was 31%, of which 25% occurred in hospital. All patients were treated conservatively with intravenous antibiotics alone, and none underwent a re-intervention. Other complications of PVE occurred in 5 patients (31%) and included aortic abscess (2), decompensated heart failure (2) and cerebral embolisation (1).

**Conclusion:**

PVE in patients receiving TAVR is a relatively rare complication and has a high mortality rate.

## What’s new?

- Prosthetic valve endocarditis (PVE) in patients undergoing transcatheter aortic valve replacement (TAVR) in the Netherlands is a rare complication with incidence rates similar to those in other studies.
- The incidence of PVE post-TAVR was similar to that of PVE after conventional surgical prosthetic valve implantation.
- PVE post-TAVR in the Netherlands was associated with a high in-hospital mortality rate, comparable with the findings of previous nationwide studies.

## Introduction

Transcatheter aortic valve replacement (TAVR) is increasingly being used as an alternative treatment to conventional surgery in patients with severe aortic stenosis who are considered to be inoperable or at high or intermediate surgical risk, recently even with beneficial results in low-risk patients [1–5]. One of the most feared complications after TAVR or surgical valve implantation is prosthetic valve endocarditis (PVE) [6, 7]. Known risk factors for PVE are advanced age, renal impairment, diabetes mellitus, chronic obstructive pulmonary disease (COPD) and residual aortic regurgitation following valve surgery [8, 9]. PVE is a rare complication, with reported first-year incidences of 0.3–1.8% after surgical aortic valve implantation (SAVR) [2, 6, 10–13], associated with high morbidity and mortality rates [10, 13]. For TAVR these incidences vary from 0.57 to 3.1%, based on small studies and a few larger nationwide registries [9, 13–17]. With the shift of TAVR indication towards lower-risk patients, it is of particularly interest how the incidence and outcome of PVE post-TAVR differs from post-SAVR. This research was performed to gain insight into the incidence and outcome of PVE after TAVR in the Netherlands.

## Materials and methods

A multicentre retrospective registry study was performed in eight centres in the Netherlands performing TAVR, including all patients who underwent TAVR in these centres in the period 2009–2017. Data were extracted using the in-hospital TAVR registry of each participating centre.

The national insurance database (*Diagnose Behandel Combinatie*) was screened for the diagnosis of infective endocarditis (IE) in all post-TAVR patients. This database registers all ambulatory diagnoses and hospital admissions with main discharge diagnoses (primary and secondary) according to the International Statistical Classification of Disease and Related Health Problems (ICD-10). Every patient in the Netherlands has a unique patient identification number, which can be used for screening diagnoses in the insurance database. Patient identification numbers of all TAVR patients were screened for IE as primary or secondary diagnosis in the insurance database, using the IE-specific ICD-10 codes I33.0, I33.9, I38 and I39. Patients who were assigned one of the mentioned ICD-10 codes after the TAVR procedure, who were in hospital >1 week or who died before discharge were suspected of having IE. The data of these patients were checked for correctness of the diagnosis of IE, using the modified Duke criteria [18, 19]. Patients with possible or definite IE, based on the modified Duke criteria, were screened for the presence of PVE using patient records.

Patients with PVE were analysed for age, gender, valve type and size, date of PVE diagnosis, organism, concomitant IE of a non-TAVR valve, presence of a cardiac implantable electronic device (CIED), concomitant IE of a CIED, re-intervention (i.e. re-TAVR, conventional surgery, conservative treatment with antibiotics or palliative care), mortality and complications according to the Valve Academic Research Consortium (VARC-2) [20]. Baseline characteristics of patients with PVE were compared with those of patients without PVE. A sample population of 848 patients without PVE and with complete baseline and hospitalisation data during transcatheter aortic valve implantation was used as a control group (confidence level of 95% with a confidence interval of 1.03, which represents a margin of error of 3%).

Complications of PVE were defined as cerebral embolisation of a vegetation, embolisation of a vegetation elsewhere, development of total atrioventricular block, root abscess, valve destruction with severe aortic regurgitation (AR) and decompensated heart failure due to AR. Investigators had the possibility to add free text remarks to the database to specify certain aspects that were not covered by the database. Left ventricular function was determined using echocardiography [21]. Renal function was determined per the KDOQI (Clinical Practice Guidelines for Chronic Kidney Disease: Evaluation, Classification, and Stratification) [22]. A renal function of class 3 and lower (glomerular filtration rate (GFR) <45) was considered to be impaired renal function. Data are shown as median ± standard deviation (SD).

Normally distributed variables are reported as mean ± standard deviation (SD) and tested with the Student’s *t*-test. Non-normally distributed variables are presented as median (interquartile range, IQR), and the Mann-Whitney U test was used to test for significant differences. Frequencies and percentages were calculated and used to express categorical variables. For distribution analysis of categorical variables, the chi-square test was used. Statistical analysis was performed using SPSS version 25 (IBM Corp., Armonk, NY, USA).

## Results

### Baseline characteristics

A total of 3968 patients who underwent TAVR between 2009 and 2017 were included in the study and screened for PVE post-TAVR. The median follow-up period was 33.5 months (IQR 22.8–45.8). Of the 3968 screened patients, 16 (0.4%) developed an episode of PVE with an incidence rate of 0.14 event per 1000 person-years. The median time to onset of PVE was 177 days (IQR 67.8–721.3). In 9 patients PVE occurred within the 1st year after TAVR (early PVE) (incidence of 0.23%) and in 7 patients (0.18%) after 1 year (late PVE).

Baseline characteristics of the PVE and control groups are shown in Tab. 1. Median age was 81.5 (IQR 68–86) years, and just over half of the patients were male (56%) in the PVE group versus 82.1 (IQR 77–86) years and 46% in the control group. Besides impaired renal function, baseline characteristics were not significantly different between the two groups. In the PVE group, a history of diabetes was observed in 5 of 16 (31%) patients, hypertension in 7 of 16 (44%) patients and impaired renal function (GFR <45 ml/min) was present in 12 of 16 (75%) patients, which differed significantly from the control group (39 ml/min, IQR 29–57 vs 59 ml/min, IQR 64–83, *p* = 0.002). None of the patients who presented with PVE after TAVR had suffered from IE prior to TAVR. Left ventricular function prior to TAVR was good (left ventricular ejection fraction >55%) in 56% (9/12), moderately impaired in 31% (5/16) and severely impaired in 13% (2/16). The affected prosthetic valves were a CoreValve CRS (Medtronic Inc., Minneapolis, MN, USA) in 25% (4/16), a Sapien XT (Edwards Lifesciences LLC, Irvine, CA, USA) in 44% (7/16), an Evolut R (Medtronic Inc.) in 13% (2/16) and a Direct Flow (Direct Flow Medical Inc., Santa Rosa, CA, USA) in 19% (3/16). The approach to implantation was transfemoral in 81% (13/16), trans-subclavian in 13% (2/16) and transapical in 6% (1/16). The mean valve diameter was 26.3 mm, ranging from 23 to 31 mm.

**Table 1:** Baseline characteristics

Number of patients	PVE *n* = 16	No PVE^a^ *n* = 848	*p*-value
Age (years), median (IQR)	81.5 (68–86)	82.1 (77–86)	0.317
Male	9 (56%)	387 (46%)	0.394
Length (cm)	169 (158–180)	168 (161–175)	0.834
Weight (kg)	78 (60–97)	73 (64-83)	0.593
Renal function (eGFR, ml/min)	39 (29–57)	59 (64–83)	**0.002**
Impaired renal function	12 (75%)	434 (51%)	0.058
Hypertension	7 (44%)	535 (63%)	0.116
Diabetes mellitus	5 (31%)	200 (24%)	0.472
History of endocarditis	0	2 (0.2%)	0.846
*TAVR prosthesis*			
First-generation CoreValve	4 (25%)	336 (40%)	
Evolut R	2 (13%)	67 (8%)	
Sapien XT	7 (44%)	216 (25%)	
Sapien 3	0	26 (3%)	
Engager	0	18 (2%)	
Direct Flow	3 (19%)	23 (3%)	
Lotus valve	0	101 (12%)	
Jena valve	0	63 (7%)	
*Left ventricular ejection fraction*			0.709
Good (>55%)	9 (56%)	560 (66%)	
Moderate (35–55%)	5 (31%)	216 (25%)	
Poor (<35%)	2 (13%)	74 (9%)	

^a^This is a selected sample population consisting of patients without PVE after TAVR

*EF* ejection fraction*, eGFR* estimated glomerular filtration rate*, PVE* prosthetic valve endocarditis*, TAVR* transcatheter aortic valve replacement

All patients had positive blood cultures, of which the most prevalent pathogen was *Enterococcus faecalis* (5/16). The other pathogens were *Staphylococcus aureus* (4/16), *Streptococcus mitis* (3/16), *Klebsiella oxytoca, Pseudomonas aeruginosa, Streptococcus gallolyticus* and a diphtheroid rod which was not defined in more detail. A possible entry point for bacteria was found in 9 patients: a gastro-intestinal (GI) entry point in 5 (4 GI tract and 1 oral entry point), an infected knee prosthesis, a femoral access site, a subclavian access site, and an inguinal abscess of the ipsilateral primary access site which developed 1 month after TAVR. Microbiological characteristics including entry point and antibiotic treatment of all patients are shown in Tab. 2.

**Table 2:** Microbiological findings and treatment in the 16 patients who developed prosthetic valve endocarditis after transcatheter aortic valve replacement (*TAVR*)

	Organism	Entry point	Antibiotic treatment	Duration of antibiotic treatment (days)
1	*Staphylococcus aureus*	Subclavian artery	Augmentin, gentamicin and after 5 days flucloxacillin	11 (patient died in-hospital)
2	Diphtheroid streptococci	Unknown	Rifampicin, vancomycin	42
3	*Enterococcus faecalis*	Unknown	Amoxicillin and ceftriaxone	42
4	*Streptococcus mitis*	Unknown	Penicillin	42
5	*Enterococcus faecalis*	Unknown	Amoxicillin	42
6	*Staphylococcus aureus*	Total knee prosthesis	Rifampicin, flucloxacillin, gentamicin	23 (patient died in-hospital)
7	*Pseudomonas aeruginosa*	Femoral access site TAVR	Ceftriaxone, ceftazidime	16 (patient died in-hospital)
8	*Streptococcus gallolyticus*	Gastro-intestinal	Penicillin, gentamicin	42
9	*Staphylococcus aureus*	Unknown	Flucloxacillin, gentamicin, rifampicin	7 (patient died in-hospital)
10	*Enterococcus faecalis*	Abscess right inguinal	Vancomycin, gentamicin	42
11	*Streptococcus mitis/oralis*	Unknown	Gentamicin, penicillin, vancomycin	42
12	*Streptococcus oralis*	Mouth	Penicillin	42
13	*Klebsiella oxytoca*	Gastro-intestinal	Unknown	Unknown
14	*Enterococcus faecalis*	Gastro-intestinal	Unknown	Unknown
15	*Enterococcus faecalis*	Gastro-intestinal	Unknown	Unknown
16	*Staphylococcus aureus*	Unknown	Unknown	Unknown

### Outcomes

Outcomes of the patients with PVE after TAVR are shown in Tab. 3. The mean (±SD) length of hospitalisation was 31.2 ± 14.6 days. In the median follow-up period, mortality was 31% (5/16); 4 patients (25%) died in hospital after the diagnosis of PVE. Three of the 9 (33%) patients with early PVE died compared with 2 of 7 (29%) in the group with late PVE.

**Table 3:** Outcomes in the 16 patients who developed prosthetic valve endocarditis (*PVE*) after transcatheter aortic valve replacement (*TAVR*)

Outcome	*n* = 16
Mortality during follow-up	5 (31%)
In-hospital mortality after diagnosis of PVE	4 (25%)
Cerebral embolisation	1 (6%)
Aortic root abscess	2 (13%)
Decompensated heart failure	2 (13%)
None	11 (69%)
Infected pacemaker	0
Concomitant mitral valve endocarditis	4 (25%)
Time to IE (days)	177 (IQR 67.8–721.3)
Patients with IE within 1 year of TAVR	9 (56%)
Mean (±SD) length of hospitalisation (days)	31.2 ± 14.6

*IE* infective endocarditis, *IQR* interquartile range

Concomitant mitral valve endocarditis was present in 25% (4/16) of patients. Of the 4 patients who developed concomitant mitral valve endocarditis, 2 (50%) died. None of the patients with a pacemaker in situ developed a device-related infection.

Complications of PVE occurred in 31% (5/16) of the patients and consisted of cerebral infarction due to embolisation of vegetation in 1 patient, aortic root abscess in 2 patients and decompensated heart failure in 2 patients. Moderate aortic valve regurgitation developed in 50% (8/16) of the patients. The initial treatment of choice was conservative, i.e. antibiotics alone in 88% (14/16) of patients, 2 of 16 patients entering a palliative care setting directly after the PVE diagnosis and 1 patient entering a palliative care setting shortly after starting antibiotic treatment. All antibiotic treatment was initiated after consultation with the attending microbiologist. None of the patients underwent a re-intervention of the TAVR prosthesis. Two patients died before assessment of aortic regurgitation could be performed.

## Discussion

This study investigated the incidence and outcomes in patients with an episode of PVE after TAVR in the Netherlands. We found an overall incidence of 0.4% (incidence rate of 0.13 event per 1000 person-years) with a higher incidence of early PVE after TAVR compared with late-onset PVE (0.23% vs 0.18%). In-hospital mortality in these patients was 25% and overall mortality 31%.

Several other studies have investigated the incidence of PVE post-TAVR, with conflicting results, and reported first-year incidences varying from 0.57 to 3.1% [2, 9, 13–17]. When comparing our results with those of previous studies, especially other nationwide registries, we observed lower incidences (cumulative and incidence rate). In the studies of Bjursten et al. (Sweden) [17] and Butt et al. (Denmark) [13], post-TAVR patients were screened in a similar way to the procedure in our study. Both reported higher cumulative first-year incidences (1.4% [17] and 2.3% [13] vs 0.23% in our study), while incidence rates were more comparable (0.16 vs 0.13 events per 1000 person-years [13]). This discrepancy in incidence can be explained by the different definitions of IE used across these studies. In some studies, only cases of PVE or definite IE, as defined by the modified Duke criteria, were included, whereas other studies also include possible IE or all patients with IE. We examined only patients with PVE, resulting in a lower incidence as compared to that in other studies using other definitions. Other possible explanations could be a difference in predisposing factors and risk profiles or the small number of patients in most studies.

With the ongoing shift in TAVR indication from high-risk towards intermediate- to low-risk patients, the comparison between PVE after SAVR versus TAVR becomes even more interesting. Reported incidences of PVE post-SAVR are low and comparable to those after TAVR, with incidences varying from 0.3 to 1.8% per year [2, 6, 11–13], while bioprosthetic surgical valves were more prone to PVE than their mechanical equivalents [12].

PVE has been associated with a grave outcome and high mortality rates. In-hospital mortality rates reported from previous studies ranged from 11% to even 67% [13, 15, 17, 23–25]. In our study, we observed an in-hospital mortality rate of 25%. In contrast with these findings, SAVR patients who develop PVE seem to have better outcomes, with reported in-hospital mortality rates of 14–25% [10, 13]. The more advanced age, frailty and presence of more co-morbidities in TAVR patients compared with SAVR patients could explain this difference in mortality.

All patients who developed PVE following TAVR were treated conservatively with antibiotics, and even after prolonged antibiotic treatment the attending physicians did not consider any surgical re-intervention (SAVR or re-TAVR). Even patients having a *Staphylococcus aureus* PVE (25%), known for its high complication rate, did not undergo re-intervention. This finding may be explained by the high to prohibitive surgical risk of this frail patient population added to the surgical difficulties of transcatheter aortic valve explantation. Re-intervention in patients with PVE after TAVR has been reported previously. However, it was still associated with high in-hospital mortality rates [9]. The number of re-interventions in patients with PVE after TAVR will probably rise in the future, due to the shift towards younger and lower-risk patients with fewer co-morbidities. The role of valve-in-valve TAVR in patients with PVE is still unclear, and future research investigating the feasibility and safety of valve-in-valve TAVR in treating PVE is necessary.

There were differences in baseline characteristics (i.e. more frequent impaired renal function in the PVE group) between the two groups. We were, however, unable to test for independent risk factors, due to the low number of patients with PVE. On the other hand, renal impairment was previously described as a risk factor for PVE post-TAVR [9, 17]. Other identified risk factors include older age, male sex, diabetes, COPD, vascular complications and residual aortic regurgitation post-TAVR [9]. PVE was more often observed in patients with a first-generation TAVR valve (i.e. Sapien XT, Direct Flow and CoreValve), compared to newer-generation TAVR devices. The use of the newer-generation devices has reduced procedural complications such as residual aortic regurgitation and vascular complications, which may have resulted in a lower risk of PVE. However, more data on the incidence of PVE related to device type are necessary.

The most frequent entry point for bacteria was the GI tract, but second came the TAVR access site, with one patient developing PVE just weeks post-TAVR. Thus, despite TAVR being a minimally invasive procedure, there is still a chance of it being the source of potentially life-threatening infections. A temporary pacing lead may also be an entry point for bacteria into the bloodstream. *Staphylococcus aureus* was the cause of PVE in 25% of the patients, which was comparable with the findings of other studies [17, 23].

### Limitations

This is a retrospective study with inherent limitations. The number of patients with actual PVE is small, limiting statistical analysis. As this study relies on an insurance database for identification of patients with IE, it depends on correct registration of these data. It is possible that patients were missed due to inappropriate code registration on hospital admission. The relatively short follow-up of this patient population who developed PVE after TAVR in this study makes it difficult to draw any conclusions on long-term outcome. PVE is a rare disease, making prospective research very difficult. We therefore have to rely on data from retrospective studies for diagnosis, treatment and prognosis of this often-lethal disease.

## Conclusion

This was the first study investigating the incidence of PVE after TAVR in the Netherlands. The incidence of PVE after TAVR was low, but it had a grave outcome with a high mortality rate.
